# Supplementary material for: Identification of photoperiod-induced specific miRNAs in the adrenal glands of Sunite sheep (Ovis aries)
Source: Front Vet Sci. 2022 Jul 22;9:888207. doi: 10.3389/fvets.2022.888207 (PMC9354845; doi:10.3389/fvets.2022.888207)
Supplement: Supplementary file 1 [file Data_Sheet_1.ZIP › Supplementary file/miRDeep2 file.zip!/miRDeep2 file/Novel_Structure/SP42A2/Novel_500.pdf]

Provisional ID	:	NC_019467.2_21988
Score total	:	1.4
Score for star read(s)	:	-1.3
Score for read counts	:	0
Score for mfe	:	1.1
Score for randfold	:	1.6
Score for cons. seed	:	
Total read count	:	12
Mature read count	:	12
Loop read count	:	0
Star read count	:	0

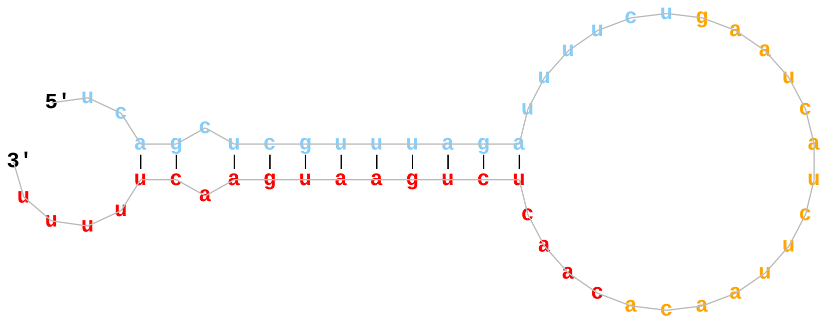

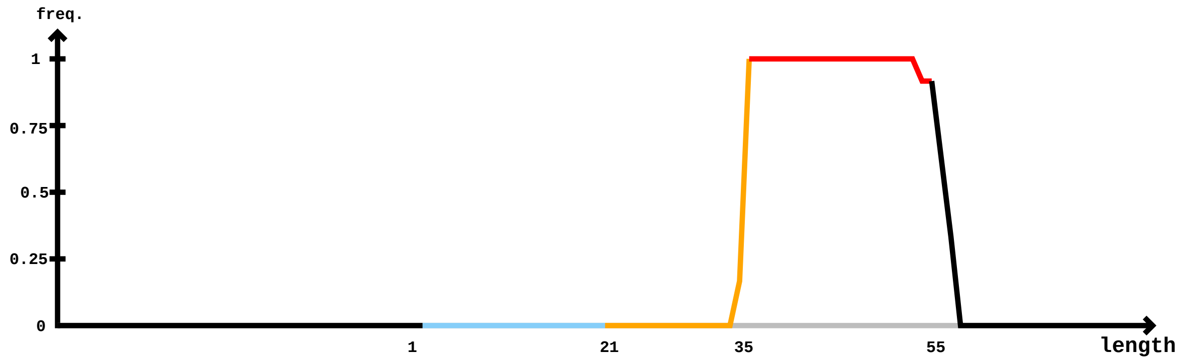


```
                                   Star                          Mature
5'- uucugagcuugguauuagugauauuggcuuggcuaaucagcucguuuagauuuucugaaucaucuuaacacaacucugaaugaacuuuuuggccagcccaaauacaagcuca   -3'   exp
    ...((((((((.((((..((.....((((.((((((..((.(((((((((........................))))))))).))..))))))))))))))))))))))))   reads   mm   sample
    ....................................................................acaacucugaaugaacuuu.........................   1       0    seq
    ....................................................................acaacucugaaugaacuuuuu.......................   1       0    seq
    .....................................................................Aaacucugaaugaacuuuuu.......................   6       1    seq
    .....................................................................caacucugaaugaacuuuuugg.....................   4       0    seq
```
